# Telemedicine for general practice: a systematic review protocol

**DOI:** 10.1186/s13643-015-0115-2

**Published:** 2015-10-05

**Authors:** Martin J. Downes, Merehau C. Mervin, Joshua M. Byrnes, Paul A. Scuffham

**Affiliations:** Centre for Applied Health Economics, School of Medicine, Menzies Health Institute Queensland, Griffith University, Logan Campus - L03, University Drive, Meadowbrook, QLD 4131 Australia

**Keywords:** Telemedicine, Telehealth, General practice, Teleconsult

## Abstract

**Background:**

The use of information technology in healthcare is fast becoming an alternative and supporting method of providing many forms of services in a healthcare and health management setting. Telephone technology is used readily to deliver services such as disease management, consultations and behaviour coaching. Telemedicine provides a promising alternative and supporting service for face-to-face general practice care. The aim of this review is to utilise a systematic review to collate evidence on the use of telemedicine as a lead in and an alternative to general practice visits.

**Methods/design:**

A systematic search of MEDLINE, CINAHL, the Cochrane Library and the International Clinical Trials Registry Platform will be performed using the search terms for the intervention (telemedicine) and the comparator (general practice) to search the databases. The systematic review aims to identify randomised control trials; however, if none are identified, an updated search will be conducted to identify lower levels of evidence. Papers will be reviewed and assessed for quality and data extracted using two reviewers; if consensus is required, a third reviewer will be consulted. If applicable, a meta-analysis of relevant outcomes will be conducted. The protocol has been reported according to the Preferred Reporting Items for Systematic Reviews and Meta-Analyses protocols (PRISMA-P) guidelines.

**Discussion:**

The intervention and comparator have the potential to provide a vast range of healthcare services to a range of diseases and health conditions. There is likely to be difficulty in identifying relevant clinical outcome measures for the patient population. A range of outcome measures will therefore be collected in the data extraction phase.

**Systematic review registration:**

PROSPERO CRD42015025225

**Electronic supplementary material:**

The online version of this article (doi:10.1186/s13643-015-0115-2) contains supplementary material, which is available to authorized users.

## Background

Telemedicine provides a promising alternative to face-to-face general practice (GP) care [1]. This is particularly true for sparsely populated regions where accesses to primary care is difficult and would require travelling long distances [2, 3]. However, whilst the published evidence has demonstrated that telemedicine is likely to be effective, there are inconsistencies in the available evidence [1].

In Australia, telemedicine is currently available for specialist services and disease management including videoconferencing by a specialist, consultant physician, telepsychiatrist, consultant occupational physician, pain medicine physician, palliative medicine physician or neurosurgeon. Telemedicine is also currently available worldwide for services such as teleradiology, behaviour management support (smoking cessation) or remote monitoring for cardiovascular disease [1]. Telephone contacts have been considered similar when used for health promotion, triage and providing long-term management [4]. Telephone consultations are currently being used in a number of countries (including the UK, the USA and Switzerland) as an alternative to a face-to-face GP consultation, and it has been suggested to provide timely care that is easily accessible [5]. Whilst there is some evidence available for telemedicine for management and monitoring in specific diseases, there is a dearth of evidence for telephone consultation as an alternative for general practice visits. A systematic review in 2010 failed to identify any publications for telemedicine as a replacement to general practice visits [1].

The aim of this study is to undertake a systematic review of the evidence on the use of telemedicine as a lead in and an alternative to general practice visits. The participants, interventions, comparators and outcomes (PICO) for this systematic review will be as follows:

- Participants: people looking to access general practice services
- Intervention: telemedicine
- Comparator: normal care (face-to-face consultation)
- Outcomes: quality-adjusted life years, hospitalisation, emergency department use, mortality, time to treatment and other relevant service outcomes

## Methods/design

A preliminary scoping search was conducted to identify terminology for the search terms and the type of studies that are likely to be available. This protocol has been reported according to the Preferred Reporting Items for Systematic Reviews and Meta-Analyses protocols (PRISMA-P) guidelines [6] (Additional file 1) and is registered with PROSPERO (CRD42015025225).

### Included study design

Systematic reviews and randomised control trials are considered the top level of evidence for decision-making, and for this reason, these will be included. However, preliminary searches have not identified systematic reviews in our population group of interest. If the systematic searches do not identify relevant studies, the review will be expanded to include cohort studies, case control studies and cross-sectional studies.

### Searches

The following sources will be searched for primary studies:

- MEDLINE, MEDLINE in process via Ovid
- CINAHL via EBSCO
- The Cochrane Library
- International Clinical Trials Registry Platform
- Checking of citation lists of included studies and relevant reviews

A combination of text words and Medical Subject Headings (MeSH) terms (for MEDLINE and the Cochrane Library) relating to telemedicine and general practice, identified in the preliminary searches, will be used. There will be no restriction on date, but publications will be restricted to those published in the English language. Sample search strategies are provided (Additional file 2), and these search terms will be adapted for each database.

The search results will be downloaded and imported into EndNote X7.3.1 (Thomson Reuters, NY). Endnote will be used to identify articles for inclusion using the predetermined eligibility criteria. Duplicate records will be identified and removed using the Endnote duplicate tool. Then, study selection will be carried out manually in three stages based on the inclusion/exclusion criteria:

1. Title screening will be carried out by one researcher and checked by another researcher for consistency.
2. Abstract reading will be carried out by two researchers and checked for consistency.
3. Full-text reading will be carried out by two researchers and checked for consistency.

Where difference between researchers occurs, agreement will be carried out either by consensus or by including a third researcher. A Preferred Reporting Items for Systematic Reviews and Meta-Analyses (PRISMA) study flow chart will be used to demonstrate the inclusion/exclusion process.

### Inclusion/exclusion criteria

Inclusion criteria:

- The studies examined telephone consultations as an alternative to direct access to general practice.
  - ○The telephone consultation was patient initiated.
  - ○The telephone consultation was carried out by a general practitioner.
- The studies followed up participants for health-related outcomes and/or healthcare utilisation.
- The studies analysed primary data.
- The studies were systematic reviews or randomised control trials (or in the case that none of these exists: The studies were cohort studies, case control studies and/or cross-sectional studies).

Exclusion criteria:

- The studies only examined telemedicine in specific disease populations.
- The studies examined only telemonitoring or the use of telemedicine for the management of disease.
- The studies examined only telemedicine used as a follow-up that was initiated by the health practitioner.
- The studies did not examine general practitioner-led telemedicine (i.e. was a study on nurse-led or specialist-led telemedicine).
- The studies outcomes were only patient satisfaction.
- The publications were narrative reviews.

### Data extraction

A standardised data extraction form will be used in the data extraction process. Data extraction will be conducted by one researcher and checked by a second researcher. Where difference occurs, these will be resolved through consensus or through a third reviewer. The data extracted from the studies will include information on the study characteristics, population baseline characteristics, the intervention, the comparator and outcomes:

- Study characteristics: author year (or other study identification), patient inclusion and exclusion criteria, study design, country, randomisation process, sample size, length of follow-up, statistical methods employed and funding source.
- Population baseline characteristics: age, gender, condition/illness, length of time since condition occurred, comorbidities, setting, i.e. general population vs. aged-care facilities and/or rural/remote vs. urban and others.
- Intervention/comparator: baseline characteristic, geographical setting, length of GP/telemedicine consult and others.
- Outcomes: There will be no restriction by outcome measures extracted, but outcomes of particular interest include hospitalisation, emergency department use, mortality, quality-adjusted life years and service outcomes, such as time waiting to be seen, time to treatment, time to secondary services, follow-up completed, compliance to GP advice and other outcome measures.

### Quality assessment (risk of bias assessment)

Critical appraisal of studies that fulfilled both the inclusion and exclusion criteria will be carried out independently by two researchers. Risk of bias for the included RCTs will be assessed using the Cochrane Collaboration’s ‘Risk of bias’ tool (Version 5.1.0.) [7]. Where any disagreements occur between the first researcher and the second, these will be marked and resolved through consensus or through a third reviewer. Where studies of other design are included, appropriate risk of bias tools will be identified and utilised for the particular type of study. The quality assessment of a study will be used to determine the strength of evidence for the outcome it represents and will be considered if a meta-analysis is conducted.

### Analysis

Meta-analysis will be conducted where appropriate if two or more similar studies provide the same outcome measures. Assessment of study heterogeneity and biases will be considered when determining if a meta-analysis is appropriate. Given the heterogeneity that is likely between the methods of delivering a telemedicine intervention, a random effects model will be used; however, if the studies are sufficiently similar, a fixed effects model may be considered. Statistical heterogeneity will be assessed using the *I*^2^ statistic [8]. Systematic differences due to publication bias will be investigated between reported and unreported findings using funnel plots and the Egger test [9].

Where meta-analysis is not possible, a narrative presentation of the study results will be provided. The systematic review and meta-analysis will be reported consistent with the PRISMA guidelines [10]. The strength of the evidence will be assessed using the Grading of Recommendations Assessment, Development and Evaluation (GRADE) guidelines to aid in the interpretation of the existing evidence and presenting recommendations for practice and future research [11].

## Discussion

Recruitment for participants into a randomised control trial for a population group that is seeking general practice care is difficult, and it is probable that the recruitment methods for the included studies in this review will vary greatly. Therefore, the results from the included studies will need to be interpreted in the context of participant recruitment, study quality and sample size. This will also have an impact on the overall results and interpretation of the systematic review. Aggregation of data may not be possible, and a narrative review may be required. The authors will need to be careful when presenting the data in a qualitative manner and incorporate the included study limitations and biases in our interpretation. However, findings from this review will be of significant importance, given the increasing use of telemedicine and access to technology in health care. Disparities in the existing evidence will be identified especially in terms of health outcomes, and this will be useful in informing future studies in this area.

## Additional files

Additional file 1:
**Preferred Reporting Items for Systematic Review and Meta-Analysis Protocols checklist.** This additional file is a completed PRISMA-P checklist for the current systematic review protocol. (DOC 84.5 KB) Additional file 2:
**Search criteria used for a systematic review in telemedicine.** The additional file includes the search terms that will be used in each database to conduct a systematic review in telemedicine. (PDF 86.5 KB)

## Abbreviations

GRADE: Grading of Recommendations Assessment, Development and Evaluation
MeSH: Medical Subject Headings
PRISMA: Preferred Reporting Items for Systematic Reviews and Meta-Analyses
PRISMA-P: Preferred Reporting Items for Systematic Reviews and Meta-Analyses protocols
