# Supplementary material for: Telemedicine for general practice: a systematic review protocol
Source: Syst Rev. 2015 Oct 5;4:134. doi: 10.1186/s13643-015-0115-2 (PMC4657341; doi:10.1186/s13643-015-0115-2)
Supplement: Additional file 2: — Search criteria used for a systematic review in telemedicine. The additional file includes the search terms that will be used in each database to conduct a systematic review in telemedicine. (PDF 86.5 KB) [file 13643_2015_115_MOESM2_ESM.pdf]

Search criteria used for a systematic review in telemedicine

Ovid MEDLINE(R) 1946 to Present with Daily Update and In-Process & Other Non-Indexed Citations

(Telemedicine.mp. OR "Tele* Consult*".mp. or "*phone* Consult*".mp. OR exp Telemedicine/ OR exp Remote Consultation/ OR Teleconsult.mp. OR Telehealth.mp. OR ehealth.mp. OR tele-health.mp. OR tele-medicine.mp.) AND (General Practice.mp. OR exp Family Practice/ OR exp General Practice/ OR Family Practi*.mp. OR primary health care.mp. OR exp Primary Health Care/ OR family physician) AND (systematic review*.mp. OR meta analysis.mp OR exp Meta-Analysis/ OR Randomized Controlled Trial.pt. OR exp Randomized Controlled Trials as Topic/ OR exp Randomized Controlled Trial/ OR RCT*.mp)

CINAHL Plus with Full Text

(Telemedicine OR Teleconsult OR "Tele* Consult*" or "*phone* Consult*" OR Telephone Consultation* OR Telehealth OR ehealth OR tele-health OR tele-medicine) AND (General Practice OR Family Practi* OR primary health care OR family physician) AND (systematic review OR meta-analysis OR Randomized Controlled Trial OR RCT*)

The Cochrane Library

(Telemedicine or "Tele* Consult*" or "*phone* Consult*" or Teleconsult or Telehealth or ehealth or tele-health or tele-medicine or MeSH descriptor: [Telemedicine] explode all trees or MeSH descriptor: [Remote Consultation] explode all trees) AND (General Practice or family physician or Family Practi* or primary health care or MeSH descriptor: [Family Practice] explode all trees or MeSH descriptor: [General Practice] explode all trees or MeSH descriptor: [Primary Health Care] explode all trees) AND (systematic review* or meta analysis or RCT* or Randomized Controlled Trial.pt. or MeSH descriptor: [Randomized Controlled Trials as Topic] explode all trees)
Reviews and trials

International Clinical Trials Registry Platform

(Telemedicine OR "Tele* Consult*" OR "*phone* Consult*" OR Teleconsult OR Telehealth OR ehealth OR tele-health OR tele-medicine) AND (General Practice OR Family Practi* OR primary health care OR family physician)
